# Electroacupuncture May Inhibit Oxidative Stress of Premature Ovarian Failure Mice by Regulating Intestinal Microbiota

**DOI:** 10.1155/2022/4362317

**Published:** 2022-08-30

**Authors:** Zixiang Geng, Xiaoli Nie, Lele Ling, Bingrong Li, Peng Liu, Long Yuan, Kaiyong Zhang, Te Liu, Bimeng Zhang

**Affiliations:** ^1^Department of Acupuncture and Moxibustion, Shanghai General Hospital, Shanghai Jiao Tong University School of Medicine, Shanghai 200086, China; ^2^Shuguang Hospital, Shanghai University of Traditional Chinese Medicine, Shanghai 201203, China; ^3^Shanghai Geriatric Institute of Chinese Medicine, Shanghai University of Traditional Chinese Medicine, Shanghai 200031, China; ^4^Shanghai Key Laboratory of Acupuncture Mechanism and Acupoint Function, Department of Aeronautics and Astronautics, Fudan University, Shanghai 200433, China

## Abstract

Premature ovarian failure (POF) is the leading cause of female infertility, and there is no optimal treatment or medication available currently. For POF, electroacupuncture (EA) has been considered a promising therapeutic approach, but the mechanism for this is not clear. In this study, we explored the effects of EA (CV4, ST36, and SP6) on oxidative stress and intestinal microbiota of high-fat and high-sugar- (HFHS-) induced POF mice. The development of mice follicles was observed by hematoxylin and eosin (HE) staining. The serum levels of estrone (E1), estrogen (E2), estriol (E3), and 21-deoxycortisol (21D) were measured by the HPLC-MS/MS method. The concentrations of Fe^2+^, superoxide dismutase (SOD), hydroxyl radical (·OH), glutathione (GSH), superoxide anion, and malondialdehyde (MDA) were measured by spectrophotometry. The 16S-rDNA sequencing was used to measure many parameters related to the host gut bacteriome and mycobiome composition, relative abundance, and diversity. mRNA expression levels of ferroptosis-related genes were determined by RT-qPCR. After 4 weeks of EA intervention in POF mice, mature follicles were increased and the levels of the sex hormone were improved. SOD activities, antisuperoxide activities, and GSH increased while MDA, ^·^OH, and Fe^2+^ decreased. In addition, EA also altered the intestinal microbiota. These results reveal that EA can effectively inhibit ovarian oxidative stress and the accumulation of Fe^2+^ in POF mice. It may be that the alteration in the intestinal microbiota is one of the potential mechanisms of EA treatment. These findings suggest that EA has clinical potential as a safe treatment for POF.

## 1. Introduction

Premature ovarian failure (POF) is a severe disorder characterized by excessive loss of ovarian oocytes and abnormalities of sex hormones [1]. In recent years, POF is becoming a common disease in the female reproductive system and according to the epidemiological survey, the incidence rate of POF is 1% in women [2]. Recent studies found that dietary and environmental factors are closely associated with the development of POF [2, 3], and a high-fat and high-sugar (HFHS) diet impairs the function of ovary and the quality of ovum [4–6]. Our previous studies have demonstrated that HFHS can induce oxidative stress and HFHS is a risk factor in the POF [7–9]. Ferroptosis is a new form of programmed cell death that is closely related to iron-dependent lipid peroxidation and reactive oxygen species (ROS) dependent [10, 11]. Under oxidative stress, phospholipids and cholesteryl esters containing polyunsaturated fatty acids (PUFAs) in cell membranes and lipoproteins are easily oxidized by the free radical-induced lipid peroxidation (LPO) process to form oxidized products. Recent studies reveal that inhibiting ferroptosis can alleviate the pathologic process of vascular calcification and atherosclerosis in vascular smooth cells and aortic endothelial cells [12, 13]. Nevertheless, the specific mechanism of HFHS and induced oxidative stress and ferroptosis remains a mystery.

The importance of the intestinal microbiota has been more and more confirmed in some in-depth studies. A few studies have demonstrated that intestinal microbiota plays a significant role in regulating host metabolism, immunity, intestinal barrier, and gut homeostasis [14]. Previous studies have proved that HFHS can alter the intestinal microbiota and affect the health of the host [15–18]. Some studies have reported that through the intestinal microbiota, HFHS influences the gut-brain axis and plays an important role in affecting the secretion of gonadal hormone levels [19]. Tan et al. found that in mice colonized with fecal microbiota from women with polycystic ovarian syndrome (PCOS), the ovarian function was altered following fecal microbiota transplantation [15]. Past studies showed that cisplatin-induced POF was associated with the intestinal microbiota [20]. However, no studies have evaluated the effects of the intestinal microbiota on HFHS-induced POF.

As a traditional Chinese medicine (TCM) therapy, electroacupuncture (EA) has a unique capability of treating POF. Some underlying mechanisms of EA for POF have been confirmed that EA could inhibit follicular loss, activate serum anti-Müllerian hormone (AMH), and enhance activation of antioxidative and antiapoptotic [21–23]. In addition, EA can improve reproductive function by reshaping and adjusting the abundance of the intestinal microbiota [24]. But whether EA can regulate the intestinal microbiota of POF remains unclear. Therefore, our study is aimed at exploring the effect of EA on oxidative stress and the relationship with the intestinal microbiota.

## 2. Methods

### 2.1. Experimental Animals

A total of 36 C57BL/6 female mice (aged 6-8 w; weighting 18 ± 2 g) were purchased from the animal laboratory center, Shanghai General Hospital, Shanghai Jiao Tong University School of Medicine (photoperiod: 12 dark/12 light; humidity: 50-70%). The mice were kept for a week of adaptive feeding. Experimental procedures involving animals were approved by the Animal Ethics Committee at the Shanghai General Hospital (2020AW126).

### 2.2. POF Model Establishment and EA Treatment

According to our previous method [25], 36 animals were randomly divided into three groups of 12 mice each: wild-type group (WT, *n* = 12), HFHS-POF group (POF, *n* = 12), and EA treatment group (EA, *n* = 12). The POF group and EA group were fed a high-fat diet (8 g/kg) and administered 200 *μ*L of 30% lactulose by gavage once daily for 8 weeks, while WT group mice were fed the daily standard maintenance diet and received 200 *μ*L of saline once daily for 8 weeks. Then, EA for 4 consecutive weeks was administered at Guanyuan (CV4), bilateral Zusanli (ST36), and Sanyinjiao (SP6) every 2 days in the EA group. These traditional acupuncture points were selected and manipulated according to previous clinical studies [26, 27]. The diameter of the needles (Zhong Yan Tai He, Beijing, China) was 0.18 mm, and the length of the needles was 13 mm. Sterile acupuncture needles were inserted to a depth of 2–5 mm at CV4, ST36, and SP6. By twisting the handles, a needling sensation is generated, which is then connected to an SDZ-V electronic acupuncture instrument (Hua Tuo, Suzhou, China). Afterward, the mice were stimulated with continuous waves of 1–3 Hz frequency and intensity of 0.1–1 mA. The POF group and WT group mice did not have an operation.

Anesthetized with isoflurane (3%) and sacrificed by cervical dislocation, mice were sacrificed at the end of the experiment. The cessation of heartbeat and respiration confirmed the death. After death was confirmed, samples of blood and plasma were collected and centrifuged at 3000 r/min for 15 minutes. After the ovarian tissue was removed from the mice, it was stored at -80°C until it was analyzed.

### 2.3. High-Performance Liquid Chromatography-Mass Spectrometry/Mass Spectrometry (HPLC-MS/MS)

According to our previous method [25], a 250 *μ*L sample (calibration working solution or plasma) was mixed with 25 *μ*L working solution and 250 *μ*L methanol, and the mixture was rotated for 2 min. Then, 250 *μ*L of deionized water was added and the mixture was shaken for 1 min. After centrifugation at 10,000 × g for 5 min, the mixture was transferred to a hydrophilic-lipophilic balance (HLB) elution plate and extracted several times. With the column temperature maintained at 35°C, the optimal mass spectrometric parameters were as follows: capillary voltage 3.56 kV, cone voltage 50 V, source temperature 150°C, desolvation temperature 600°C, cone gas flow 150 L/h, desolvation gas flow 1000 L/h, and collision gas flow 0.15 mL/min. The filtrate was analyzed by high-performance liquid chromatography- (HPLC-) mass spectrometry/mass spectrometry (MS/MS).

### 2.4. Hematoxylin and Eosin (HE) Staining

In brief, Beyotime Biotechnology Co., Ltd. (China), provided the reagents and materials to fix tissues using 4% paraformaldehyde at 37°C for 12 hours first [28]. An approximately 5 *μ*m thick frozen section of tissue was prepared, fixed with 95% anhydrous ethanol for 2 minutes, hematoxylin-stained for 5 minutes, and differentiated in a differentiation solution for 2 minutes. After soaking in weak ammonia for 3 minutes, the slices were washed with deionized water for 5 minutes, stained with eosin for 5 minutes, and washed with deionized water for 5 minutes. After soaking in 70%, 80%, and 90% ethanol solutions for 1 min, they were washed twice with anhydrous ethanol for 1 min and then in xylene for 1 min before being embedded in neutral sesame oil.

### 2.5. Masson Staining

The Masson trichrome staining technique, using collagen fibers as a marker, was used on all ovary sections. Muscle fibers, red blood cells, and cytoplasm were stained red, while nuclei were stained black. Using ImageJ software, the degree of fibrosis of the ovary was calculated under a magnification of 400x.

### 2.6. Oxidative Stress Marker Assay

According to our previous method [29], a tissue on ice was used to lyse the samples for two hours with lysis buffer. Following the instructions in the assay kits (Nanjing Jiancheng Bioengineering Institute Co., Ltd., Nanjing, China), Fe^2+^, hydroxyl radical (·OH), antisuperoxide activities, superoxide dismutase (SOD), malondialdehyde (MDA), and glutathione (GSH) were detected, respectively. The OD value was measured using PowerWave XS (BIO-TEK, USA), and the content of Fe^2+^(intra-assay coefficient of variation was 3.9%, interassay coefficient of variation was 6.54%, and the lowest detectable concentration was 0.05 mg/L), ^·^OH (intra-assay coefficient of variation was 3%, interassay coefficient of variation was 6.95%, and the lowest detectable concentration was 10.1 U/mL), antisuperoxide activities (intra-assay coefficient of variation was 2.5%, interassay coefficient of variation was 5.21%, and lowest detectable concentration was 0.5 U/L), SOD (intra-assay coefficient of variation was 5.5%, interassay coefficient of variation was 3.32%, and the lowest detectable concentration was 0.5 U/mL), MDA (intra-assay coefficient of variation was 3.5%, interassay coefficient of variation was 4.11%, and the lowest detectable concentration was 0.5 nmol/mL), and GSH (intra-assay coefficient of variation was 2.8%, interassay coefficient of variation was 5.32%, and the lowest detectable concentration was 0.1 *μ*mol/L) was calculated.

### 2.7. 16S-rDNA High-Throughput Sequencing

As described in previous studies [30, 31], fresh fecal samples were collected during the last 5 days of the experiment to analyze the gut microbiota. Bacterial genomic DNA was extracted from frozen samples stored at -80°C. The V3 and V4 regions of the 16S-rDNA gene were amplified by PCR using specific bacterial primers (*F* primer: 5′-ACTCCTACGGGAGGCAGCA-3′; *R* primer: 5′-GGACTACHVGGGTWTCTAAT-3′)[32]. High-throughput pyrosequencing of the PCR products was performed on an Illumina MiSeq platform at Biomarker Technologies Co. Ltd. (China). The raw paired-end reads from the original DNA fragments were merged using FLASH32 and assigned to each sample, according to the unique barcodes. QIIME (version 1.8.0) UCLUST software was used based on 97% sequence similarity. The tags were clustered into operational taxonomic units. The alpha diversity index was evaluated using Mothur software (v.1.30). ASVs and OTUs were derived from high-quality reads. Based on feature analysis, taxonomic classifications were processed, generating diagrams such as composition distribution bar graphs and heatmaps for species abundance by the level of phylum, class, order, family, genus, and species, as well as taxonomic tree and phylogenetic tree at the genus level. Alpha diversity analysis (study on species diversity within a single sample): Ace, Chao1, Shannon, and Simpson indexes of each sample were calculated at a rate of 97% similarity and generated dilution curve and rank abundance curve. In beta diversity analysis (study of differences in species diversity (composition and structure of communities) between samples), based on distance matrix, UPGMA tree, NMDS analysis, and sample clustering heatmap, PCA and PCoA plots of samples (with grouping information) and boxplot based on multiple distances were obtained. Biomarkers with statistical significance were identified by differential analysis between groups. Correlation analysis was used to study interactions between microbial communities and environmental factors. Based on the functional prediction, phenotypes, gene functions, and sample abundances were characterized and estimated. Nonparametric factorial Kruskal-Wallis sum-rank test and unpaired Wilcoxon rank-sum test were performed to identify the taxa with significantly different abundance.

### 2.8. Statistical Analysis

Each experiment was performed at least three times; data are presented as mean ± standard deviation (SD) where applicable. Differences were evaluated using ANOVA (analysis of variance) or nonparametric Kruskal-Wallis test. *P* values < 0.05 were considered statistically significant.

## 3. Results

### 3.1. EA Suppressed HFHS-Induced Ovarian Damage

**Figure 1:**
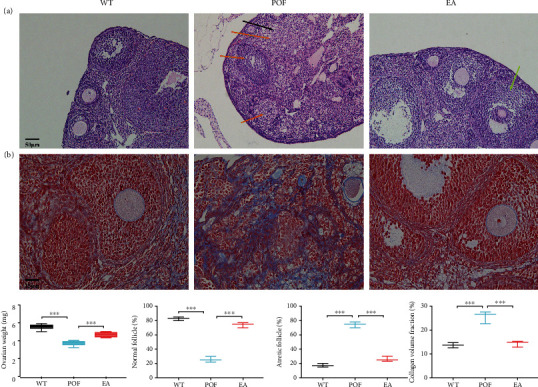
Effects of electroacupuncture (EA) in high-fat and high-sugar- (HFHS-) induced mouse model of premature ovarian failure (POF). (a) Mouse ovarian tissue stained with H&E (atresia follicles: orange arrows, fat vacuoles: black arrow, matured follicles: green arrows, ×200 magnification, 50 *μ*m); (b) Masson's trichrome stain (×400 magnification, 25 *μ*m); (c) ovarian weight (*F* = 0.01608, DFn = 2, DFd = 21, ^∗∗∗^*P* < 0.001, *n* = 8); (d) the proportion of normal follicles (*F* = 0.1556, DFn = 2, DFd = 6, ^∗∗∗^*P* < 0.001, *n* = 3); (e) the proportion of atretic follicles (*F* = 0.1556, DFn = 2, DFd = 6, ^∗∗∗^*P* < 0.001, *n* = 3); (f) collagen volume fraction (*F* = 0.4067, DFn = 2, DFd = 6, ^∗∗∗^*P* < 0.001, *n* = 3). All data were normally or approximately normally distributed.

According to our previous studies [7–9], when the mice showed low levels of estradiol (E2) and/or high levels of follicle-stimulating hormone (FSH) and ovarian pathologies presented as mature ovarian follicles disappeared and the proportion of atretic follicles significantly increased, the establishment POF mice model induced by HFHS is successful. HE staining results suggested that a large number of atresia follicles (orange arrows) and fat vacuoles (black arrow) were present in ovary tissues of the POF Compared with the POF group, the proportion of atretic follicles was significantly decreased (*P* < 0.001), while the proportion of normal follicles was significantly increased (*P* < 0.001), and matured follicles (green arrows) were found in ovary tissues of the EA group (Figures 1(a), 1(d), and 1(e)). Masson staining results suggested that the collagen in the POF group remained at a high volume fraction, and compared with the POF group (*P* < 0.001), significant suppression was observed in the EA group (Figures 1(b) and 1(f)). In addition, EA also induced recovery in ovarian weight (Figure 1(c)). The above results suggested that EA could suppress ovarian fibrosis and atretic follicle and promote follicle maturation.

### 3.2. EA Improved the Hormone Levels of POF

**Figure 2:**
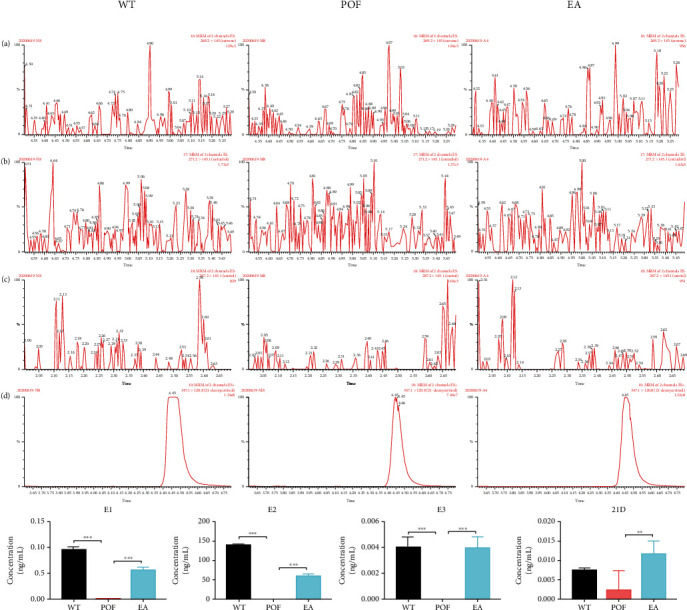
EA restores the hormone levels in POF mice. (a–d) Detailed results of HPLC–MS analysis; (e) estrone (E1, *F* = 2.12, DFn = 2, DFd = 9, ^∗∗∗^*P* < 0.001, *n* = 4); (f) estrogen (E2, *F* = 0.6629, DFn = 2, DFd = 9, ^∗∗∗^*P* < 0.001, *n* = 4); (g) estriol (E3, *F* = 1.5, DFn = 2, DFd = 9, ^∗∗∗^*P* < 0.001, *n* = 4); (h) 21-deoxycortisol (21D, *F* = 0.4872, DFn = 2, DFd = 9, ^∗∗^*P* < 0.01, *n* = 4). All data were normally or approximately normally distributed.

HPLC-MS/MS detection showed that compared with the WT group, estrone (E1), E2, estriol (E3), and 21-deoxycortisol (21D) were significantly lower in the POF group (*P* < 0.01). To a certain extent, these hormone levels were observed to be restored following treatment with EA (Figures 2(a)–2(h)). HPLC-MS/MS results demonstrated that the hormone levels of POF mice were improved by EA treatment.

### 3.3. EA Weakened Oxidative Stress Capacity and Ferroptosis

**Figure 3:**
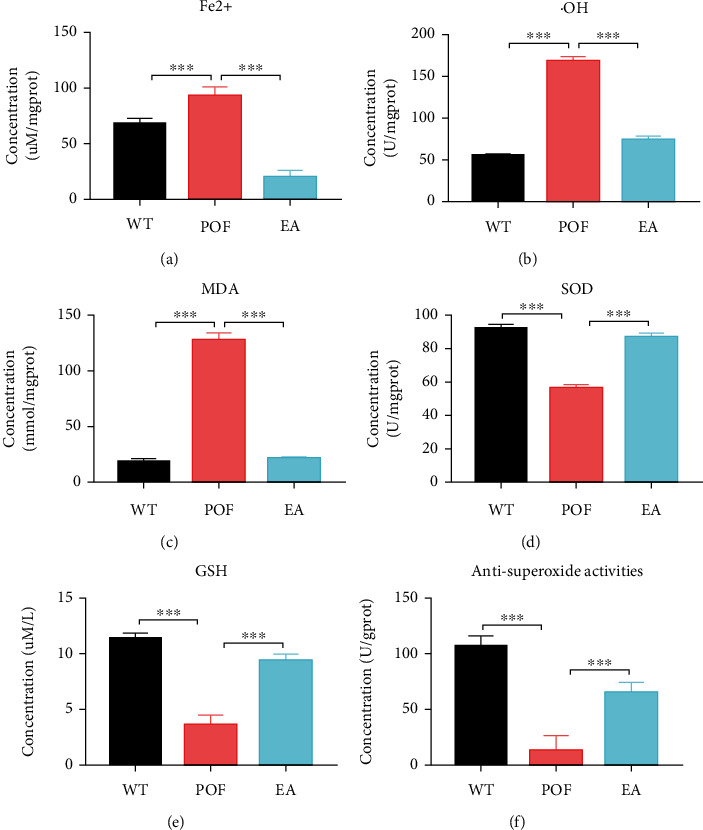
EA inhibits Fe^2+^ level and oxidative stress-related indicators. (a) Fe^2+^ (*F* = 0.05585, DFn = 2, DFd = 9, ^∗∗∗^*P* < 0.001, *n* = 4); (b) hydroxyl radical (·OH, *F* = 0.4532, DFn = 2, DFd = 9, ^∗∗∗^*P* < 0.001, *n* = 4); (c) malondialdehyde (MDA, *F* = 1.415, DFn = 2, DFd = 9, ^∗∗∗^*P* < 0.001, *n* = 4); (d) superoxide dismutase (SOD, *F* = 0.004201, DFn = 2, DFd = 9, ^∗∗∗^*P* < 0.001, *n* = 4); (e) glutathione (GSH, *F* = 0.3082, DFn = 2, DFd = 9, ^∗∗∗^*P* < 0.001, *n* = 4); (f) antisuperoxide activities (*F* = 0.1951, DFn = 2, DFd = 9, ^∗∗∗^*P* < 0.001, *n* = 4). All data were normally or approximately normally distributed.

Results suggested that the POF group had a significant increase in Fe^2+^ level compared with the WT group (*P* < 0.001, Figure 3(a)), and there is a significant decrease in the level of Fe^2+^ after EA treatment (*P* < 0.001). The result indicated that EA treatment inhibits the level of the Fe^2+^ in the ovarian tissues of mice. As compared with the WT group, GSH, antisuperoxide activities, and SOD levels were significantly decreased (*P* < 0.001, Figures 3(d)–3(f)), whereas ^·^OH and MDA levels were significantly increased (*P* < 0.001, Figures 3(b) and 3(c)). GSH, antisuperoxide activities, and SOD levels in the EA group significantly increased, while ^·^OH and MDA levels in the EA group significantly decreased, compared with the POF group (*P* < 0.001).

**Figure 4:**
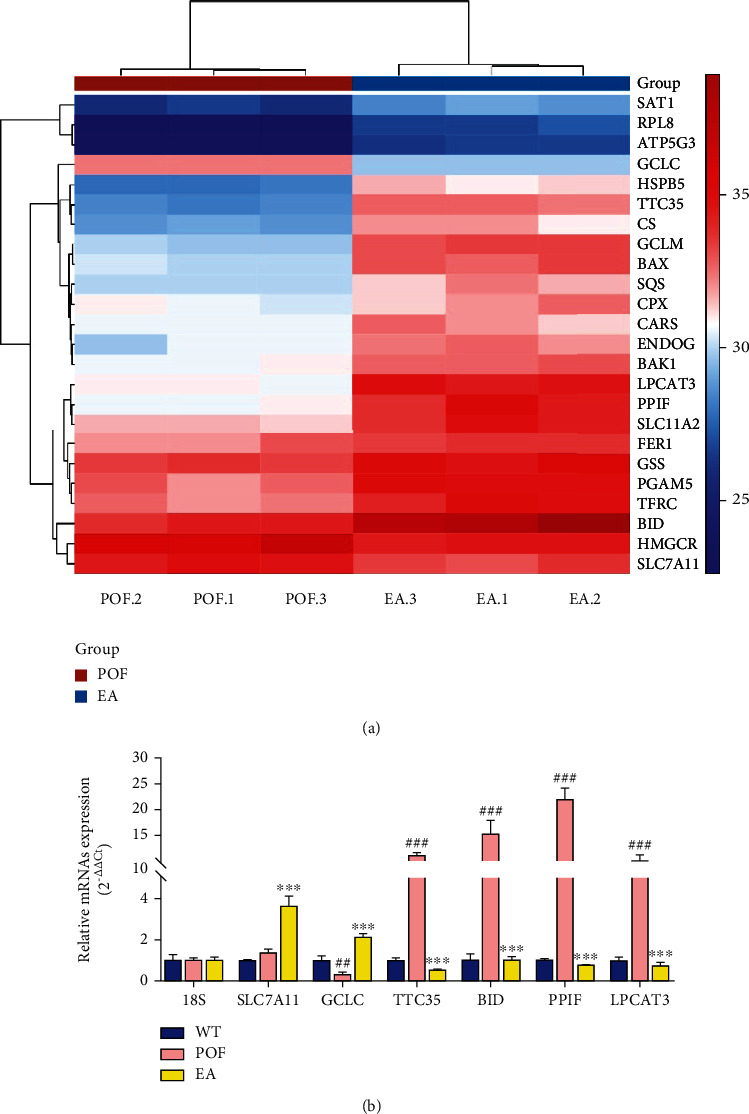
Ferroptosis is inhibited by EA treatment in ovarian tissue. (a) Heatmap analysis of ferroptosis-related genes. (b) The RT-qPCR results of SLC7A11, GCLC, TTC35, BID, PPIF, and LPCAT3 (^##^*P* < 0.01 vs. the WT group, ^###^*P* < 0.001 vs. the WT group, ^∗∗∗^*P* < 0.01 vs. the POF group, *n* = 3). All data were normally or approximately normally distributed.

The heatmap showed the ferroptosis-related genes between the POF group and the EA group were extracted, and a cluster analysis was run on the gene set (Figure 4(a)). The RT-qPCR results showed that the expression of SCL7A11 and GCLC is elevated (ferroptosis-inhibiting genes, Figure 4(b)), and the expression of TTC35, BID, PPIF, and LPCAT3 is decreased (ferroptosis-promoting genes). All the data showed that EA treatment could inhibit the capacity of oxidative stress and ferroptosis in the mouse ovary.

### 3.4. Analysis of 16S-rDNA Intestinal Microbiota Profiles in POF Mice

**Figure 5:**
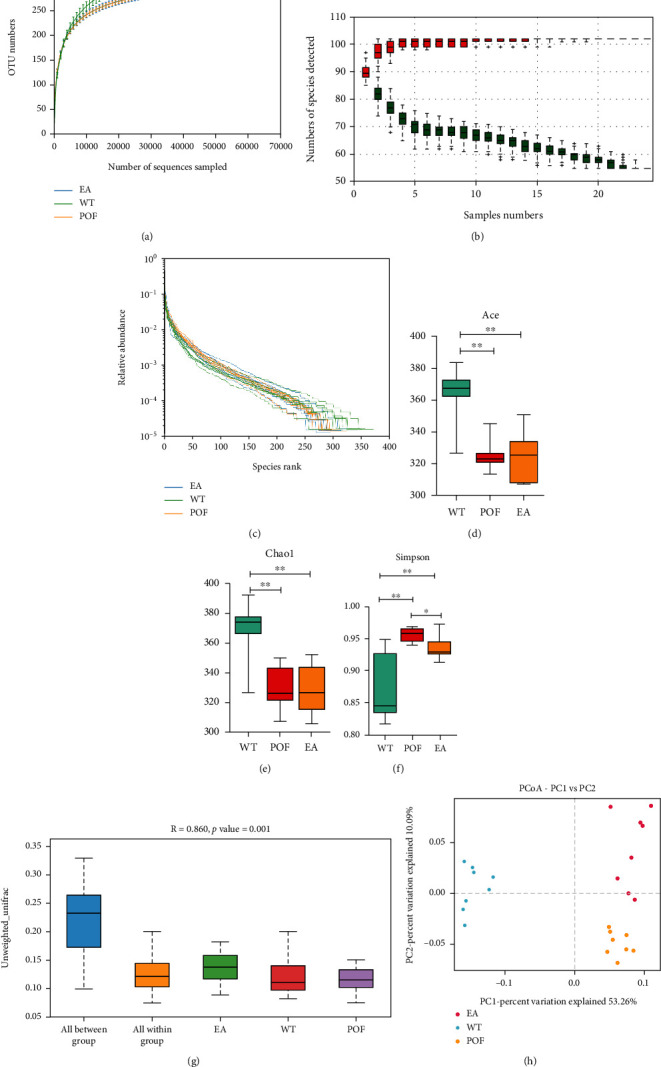
The distribution of bacteria between the three groups differed. (a–b) At the end of the species accumulation and dilution curves, the upward trend flattened which indicated that most OTUs have been captured and the sample size has been sufficiently large to cover the majority of information on microbial diversity. (c) The rank abundance analysis showed that the compositions of microorganisms within each group were similar in their richness and evenness. (d–f) Chao1 and Ace count for the species richness or the number of species presented and the Simpson index reflect species richness and community evenness (^∗^*P* < 0.05, ^∗∗^*P* < 0.01, *n* = 8). (g) Based on the PERMANOVA of differences between groups and differences within groups, it is clear that there is a significant difference between the three groups (*R* = 0.860, *P* = 0.001). (h) Principal coordinate analysis (PCoA) of unweighted UniFrac distance matrices was used to analyze the intestinal microbiota in different groups. All data were normally or approximately normally distributed.

The fecal samples from the three groups were sequenced four weeks after EA treatment. In total, 1920431 paired-end (PE) reads were generated from 24 samples. A total of 1919137 clean reads were obtained following PE quality control and assembly. The minimum and average clean reads generated for each sample were 79565 and 79964, respectively. In this study, a total of 413 OTUS with 97% similarity or greater were recognized across the 24 samples obtained. At the end of the species accumulation and dilution curves, the upward trend flattened (Figures 5(a) and 5(b)). This indicates that most OTUs have been captured, that the sample size has been sufficiently large to cover the majority of information on microbial diversity, and that increasing the sample size would uncover only a few new species. According to rank abundance analysis (Figure 5(c)), the compositions of microorganisms within each group were similar in their richness and evenness. The POF and EA groups experienced a significant reduction in *α*-diversity as compared with the WT group on the basis of the ACE (*F* = 1.1, DFn = 2, DFd = 21) and Chao1 (*F* = 0.1982, DFn = 2, DFd = 21), but Simpson (*F* = 3.658, DFn = 2, DFd = 21) in the POF and EA groups was significantly increased (*P* < 0.01). In addition, there is a significant difference in the EA group (*P* < 0.05), compared with the POF group (Figures 5(d)–5(f)). According to these findings, POF and EA significantly reduce the richness of intestinal microbiota but increase the distribution of the abundance of bacteria species. Based on the PERMANOVA of differences between groups and differences within groups (*R* = 0.860, *P* = 0.001), it is clear that there is a significant difference between the three groups (Figure 5(g)). Then, we carried out a principal coordinate analysis (PCoA) of unweighted UniFrac distance matrices. Principal component 1 (PC1) explained 53.26% of the variance, and principal component 2 (PC2) explained 10.09% (Figure 5(h)). Microbial differences were evident between the three groups, which suggested that the distribution of bacteria between the three groups differed.

### 3.5. The Impact of EA Treatment on the Intestinal Microbiota of POF Mice

**Figure 6:**
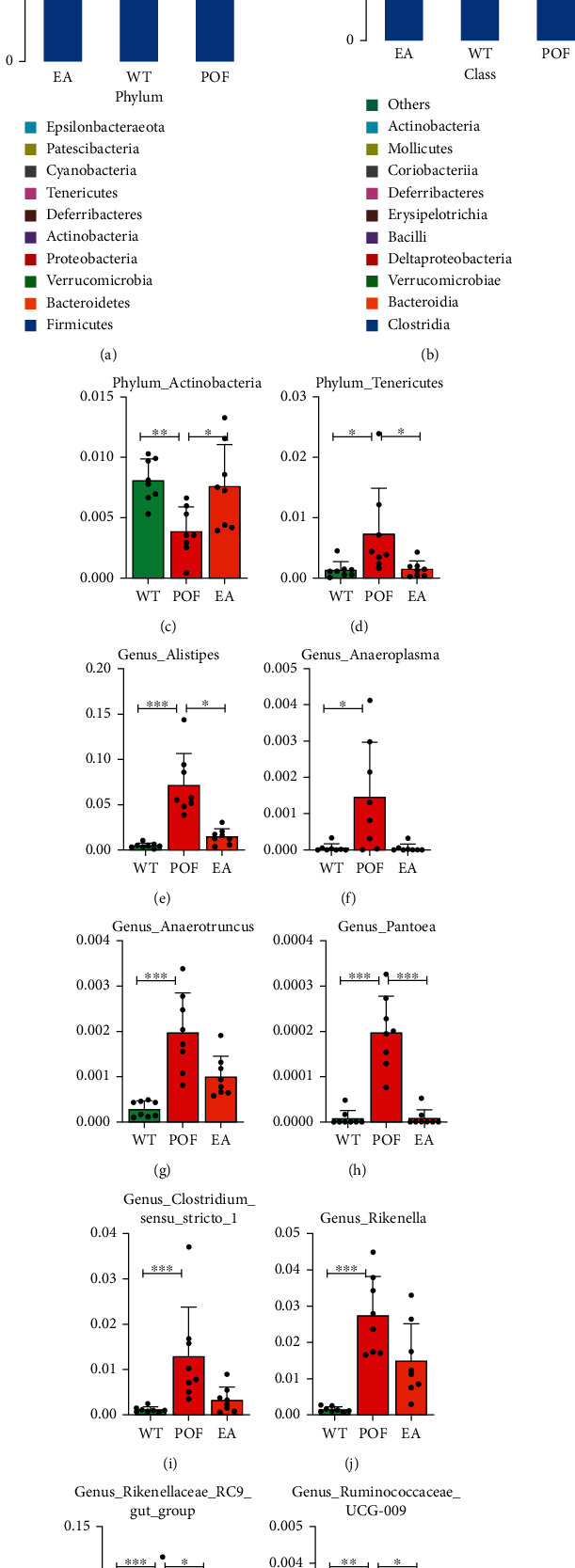
The impact of EA on intestinal microbiota of POF mice. (a) Relative abundance of microbial communities at the phylum level (*n* = 8). (b) Relative abundance of microbial communities at the class level (*n* = 8). (c–d) Relative abundance of bacteria at the phylum level (^∗^*P* < 0.05, ^∗∗^*P* < 0.01, *n* = 8). (e–i) Relative abundance of bacteria at the genus level (^∗^*P* < 0.05, ^∗∗^*P* < 0.01, ^∗∗∗^*P* < 0.001, *n* = 8). All data were normally or approximately normally distributed.

According to the phylum level microbial compositions (Figure 6(a)), *Firmicutes* and *Bacteroidetes* in the WT and EA group accounted for less than 80% of the entire microbial community, whereas those in the POF group were more than 80%. At the class level (Figure 6(b)), *Clostridia* and *Bacteroidia* in the WT and EA group accounted for less than 60% of the entire microbial community, whereas those in the POF group were more than 70%. At the phylum level, compared with the WT group, the relative abundance of *Actinobacteria* significantly decreased (*F* = 1.722, DFn = 2, DFd = 21, *P* < 0.01; Figure 6(c)) and *Tenericutes* significantly increased (*F* = 2.344, DFn = 2, DFd = 21, *P* < 0.05; Figure 6(d)) in the POF group. Compared with the POF group, the relative abundance of *Tenericutes* significantly decreased after EA treatment (*P* < 0.05), while the relative abundance of *Actinobacteria* increased (*P* < 0.05). At the genus level, compared with the WT group, there was a significant increase in the relative abundance of *Alistipes* (*F* = 3.604, DFn = 2, DFd = 21, *P* < 0.001; Figure 6(e)), *Anaeroplasma* (*F* = 11.33, DFn = 2, DFd = 21, *P* < 0.05; Figure 6(f)), *Anaerotruncus* (*F* = 5.379, DFn = 2, DFd = 21, *P* < 0.001; Figure 6(g)), *Pantoea* (*F* = 6.956, DFn = 2, DFd = 21, *P* < 0.001; Figure 6(h)), *Clostridium_sensu_stricto_1* (*F* = 3.541, DFn = 2, DFd = 21, *P* < 0.001; Figure 6(i)), *Rikenella* (*F* = 3.541, DFn = 2, DFd = 21, *P* < 0.001; Figure 6(j)), *Rikenellaceae_RC9_gut_group* (*F* = 11.42, DFn = 2, DFd = 21, *P* < 0.001; Figure 6(k)) and *Ruminococcaceae_UCG-009* (*F* = 1.625, DFn = 2, DFd = 21, *P* < 0.01; Figure 6(i)) significantly increased in the POF group. Compared with the POF group, the relative abundance of *Alistipes* (*P* < 0.05), *Pantoea* (*P* < 0.001), *Rikenellaceae_RC9_gut_group* (*P* < 0.05), and *Ruminococcaceae_UCG-009* (*P* < 0.05) significantly decreased after EA treatment.

### 3.6. The Correlations between the Intestinal Microbiota and Oxidative Stress-Related Indicators

**Figure 7:**
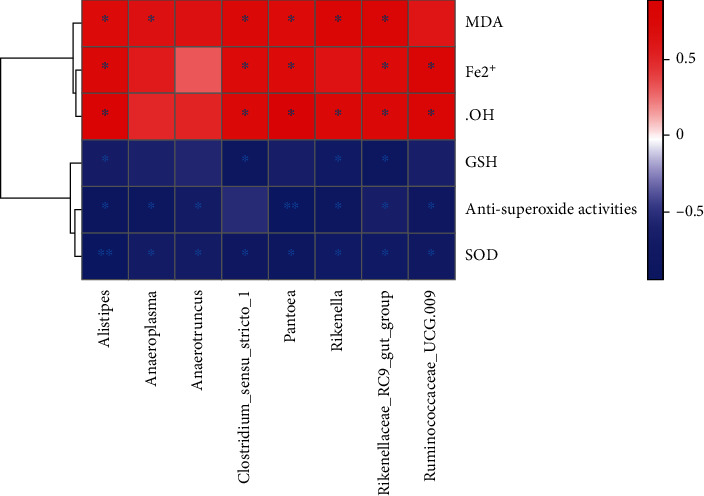
A heatmap of correlation between changes in the intestinal microbiota and changes in Fe^2+^ and oxidative stress-related indicators.

Correlation heatmap analysis was applied to assess the association between intestinal microbiota and ferroptosis-related indicators, such as Fe^2+^ and oxidative stress indicators. According to this heatmap, *Alistipes*, *Anaeroplasma*, *Anaerotruncus*, *Clostridium_sensu_stricto_1*, *Pantoea*, *Rikenella*, *Rikenellaceae_RC9_gut_group*, and *Ruminococcaceae_UCG-009* were correlated negatively with GSH, SOD, and antisuperoxide activities and positively with Fe^2+^, MDA, and ^·^OH in addition to few indicators. The analysis of the Spearman correlation indicated that there is a potential link between intestinal microbiota and oxidative stress-related indicators in ovarian tissues (Figure 7).

## 4. Discussion

Our past work demonstrated that POF was a result of pathological ovarian aging and it had very close contact with HFHS-induced oxidative stress [7–9]. Currently, EA is an effective strategy for the treatment of POF. However, the mechanism remains unknown. In our study, we showed that the effects of EA may be related to the intestinal microbiota. Here, this improvement of oxidative stress and reduction of free iron (Fe^2+^) in POF mice were considered as an effect of EA on the regulation of intestinal microbiota.

HFHS diet could not only increase the risk of obesity but also severely affect ovarian function and oocyte quality [8]. Our data showed that EA could effectively alleviate ovarian weight loss, atretic follicles, and abnormal secretion of estrogens. This is consistent with previous research [22, 23]. In addition, HFHS diet could induce oxidative stress in the female reproductive system [7–9]. Oxidative stress was defined as an imbalance between ROS production and the antioxidant capacity of cell [33]. A major function of SOD and GSH is to scavenge ROS and protect against oxidative stress by maintaining the redox balance of the cell. MDA, ^·^OH, and superoxide anion are important biomarkers of oxidative stress and are widely used as indicators of oxidative injury [34]. ·OH is generated through Fenton reactions when H_2_O_2_ reacts with Fe^2+^, resulting in lipid peroxidation DNA damage [35]. Our study suggested that SOD activities, antisuperoxide activities, and GSH decreased while MDA, ^·^OH, and Fe^2+^ increased in POF mice. These indicated that the HFHS diet causes oxidative stress damage and lipid peroxidation and reduces antioxidant enzyme activities. After treatment, we found that EA alleviated ovarian oxidative stress injuries. Moreover, EA also promoted the expression of SCL7A11 and GCLC (ferroptosis-inhibiting genes) and inhibited TTC35, BID, PPIF, and LPCAT3 (ferroptosis-promoting genes). The above results suggested that EA could inhibit oxidative stress and ferroptosis.

Several studies demonstrated that HFHS can alter the composition of the intestinal microbiota, enhance intestinal permeability, elevate plasma lipopolysaccharide and endotoxin level, and evoke inflammatory response [36–38]. HFHS increased not only intestinal oxidative stress but also other organs by inducing disturbance of the intestinal microbiota [39]. In addition, HFHS could elevate the serum levels of Fe^2+^ and lead to an increase of lipid peroxidation [40]. Hence, we speculate that the elevated circulating endotoxin and Fe^2+^ levels induced by intestinal microbiota disturbance may be associated with oxidative stress in the ovaries.

Past studies have suggested that EA could modulate intestinal microecology and protect the intestinal barrier [41–43]. Zhang et al. showed that EA regulated the metabolic disorders and improved reproductive function in this PCOS-like rat model by adjusting intestinal microbiota [24]. Similarly, we found that EA has positive effects on both restoration of the percentage of normal follicles and improvement of intestinal microbiota disturbance. EA increased the diversity of the intestinal microbiota and Simpson index and decreased the relative abundance of the intestinal microbiota, ACE index, and Chao1 index. At the phylum level, the relative abundance of the *Actinobacteria* significantly increased and *Tenericutes* decreased after EA treatment. At the genus level, EA resulted in a significant decrease in the abundance of *Alistipes*, *Anaeroplasma*, *Anaerotruncus*, *Pantoea*, *Clostridium_sensu_stricto_1*, *Rikenella*, *Rikenellaceae_RC9_gut_group*, and *Ruminococcaceae_UCG-009*. In our analysis, we found that these microbiomes were inversely associated with improvement of oxidative stress injury and reduction of Fe^2+^ levels in EA treatment. This further validates our speculation that EA could inhibit oxidative stress by adjusting intestinal microbiota.

Some limitations in the study should be acknowledged. First, this study shows that EA could alter the intestinal flora and inhibit ovarian oxidative stress, and there is a certain connection between them. However, there is no direct evidence to suggest how intestinal flora participates in the inhibition of oxidative stress. We speculate that metabolic products produced by the specific microbiome may inhibit oxidative stress or changes in the intestinal flora may disrupt Fe^2+^ homeostasis. It will need further study. Second, we opted for the most commonly used points (CV4, ST36, and SP6) of POF in this study. There were many studies that has explored the neurobiological basis of ST36, but other points have not been confirmed [44, 45]. The combined effects of these points are also not clear. Hence, these potential associations need further exploration.

## 5. Conclusion

In summary, EA can effectively inhibit ovarian oxidative stress and the accumulation of Fe^2+^ in POF mice. It may be that the alteration in intestinal microbiota is one of the potential mechanisms of EA treatment. Our findings demonstrate the therapeutic potential of EA for the treatment of POF, and it is worthy of further clinical exploration.

## Data Availability

The processed data are available from the corresponding authors upon request.
